# Isolation, characterization, and biocontrol potential of a novel lytic phage vB_VpaS_R27Z against *Vibrio parahaemolyticus* for mitigating seafood contamination

**DOI:** 10.1016/j.crfs.2026.101602

**Published:** 2026-10-22

**Authors:** Hanxi Xiao, Muhammad Imran Taj, Biao Zhu, Chaoyin Xia, Mingfu Wang, Bu Xu, Rui Zhang

**Affiliations:** a Archaeal Biology Center, Synthetic Biology Research Center, Shenzhen Key Laboratory of Marine Microbiome Engineering, Key Laboratory of Marine Microbiome Engineering of Guangdong Higher Education Institutes, Institute for Advanced Study, Shenzhen University, Shenzhen, 518055, China; b Shenzhen Key Laboratory of Food Nutrition and Health, College of Chemistry and Environmental Engineering, Shenzhen University, Shenzhen, 518060, China

**Keywords:** *Vibrio parahaemolyticus*, Lytic bacteriophage, Biocontrol agent, Antibacterial activity, Seafood

## Abstract

*Vibrio parahaemolyticus* is a critical seafood-borne pathogen that poses substantial risks to food safety and public health. Effective mitigation of this bacterium in fresh and minimally processed seafood remains challenging, since conventional antimicrobial treatments induce antibiotic resistance and degrade the sensory quality of seafood products. As natural antimicrobial agents, lytic bacteriophages represent a targeted alternative to antibiotics for suppressing *V. parahaemolyticus* in seafood. In this study, we isolated and characterized a novel lytic bacteriophage, vB_VpaS_R27Z (R27Z), targeting *V. parahaemolyticus*, and further assessed its biocontrol potential against this pathogen in seafood matrices. Phage R27Z exhibited siphoviral morphology, featuring an icosahedral capsid and a long non-contractile tail. It possesses a short latent period of 20 min and a burst size of approximately 58 PFU per infected cell, reflecting its high lytic replication efficiency. R27Z is stable at a broad temperature range of 4°C to 50°C and a pH range from 5 to 10, suggesting tolerance to conditions relevant to seafood handling and storage. Genomic analysis revealed that the genome size of R27Z was 51,052 bp and it contained 64 open reading frames. Notably, no genes associated with lysogeny, bacterial virulence, or antibiotic resistance were detected within its genome, which is essential for food applications. In vitro growth inhibition assays demonstrated that R27Z inhibited *V. parahaemolyticus* growth at multiplicities of infection (MOIs) of 0.01, 0.1, 1, 10, and 100, with the strongest early-phase inhibition observed at MOIs of 10 and 100. Furthermore, R27Z markedly suppressed *V. parahaemolyticus* on both fish flesh and fish skin, with maximum reductions of 3.62 log_10_ and 2.84 log_10_ units at an MOI of 1 alongside rising recoverable phage titers throughout treatment, confirming host-supported replication in these food matrices. Collectively, these results highlighted that R27Z could act as a potential alternative natural biocontrol agent against *V. parahaemolyticus* in seafood products.

## Highlights

• A novel lytic phage vB_VpaS_R27Z was isolated and characterized for biocontrol of *Vibrio parahaemolyticus.*
• Phage R27Z has a short latent period, a high burst size and excellent tolerance across broad temperature and pH ranges.
• Genomic analysis confirmed R27Z carries no lysogenic, virulence or antibiotic resistance-related genes.
• R27Z exhibits efficient in vitro growth inhibition against d at multiple MOIs.
• Phage R27Z achieves significant pathogen reduction in fish flesh and skin, demonstrating great seafood biocontrol potential.

## Graphical abstract

**Figure:**
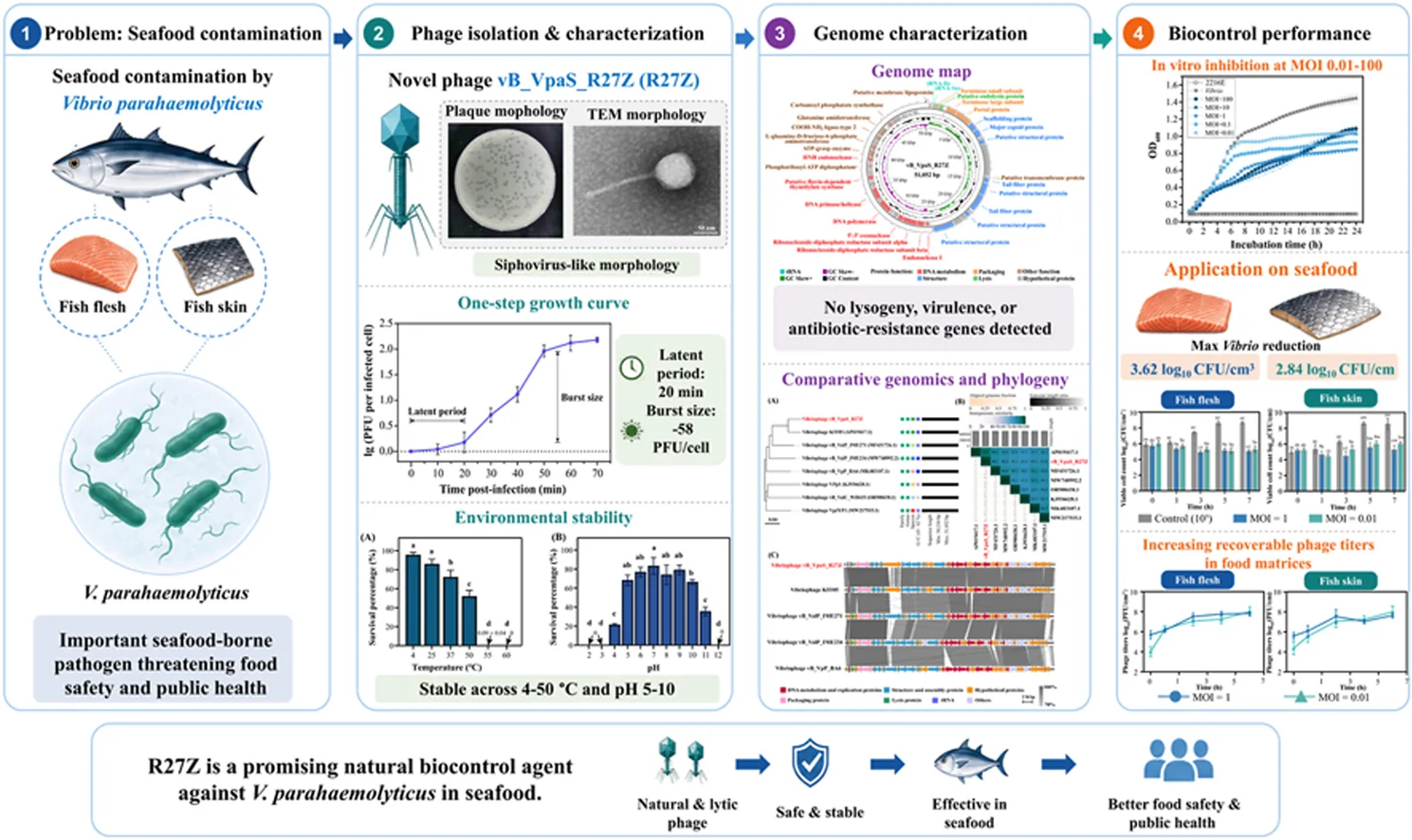

## 1 Introduction

Seafood has become increasingly important in meeting the global demand for safe and nutritious animal protein. Fish, shrimp, and other seafood products provide high-quality protein, long-chain polyunsaturated fatty acids, minerals, and vitamins, and their contribution to human diets continues to rise with the expansion of aquaculture. In 2022, aquaculture surpassed capture fisheries as the major source of aquatic animals for human consumption, underscoring its growing role in global food supply (FAO, 2024). This expansion also increases the pressure on food-safety systems, because seafood can acquire microbial contaminants during farming, harvesting, transportation, processing, retail handling, and domestic preparation. Foodborne diseases remain a major public-health burden worldwide, and seafood-associated pathogens are particularly difficult to control when products are consumed raw, undercooked, or minimally processed (Brauge et al., 2024).

*Vibrio parahaemolyticus* is a halophilic Gram-negative bacterium widely distributed in marine and estuarine environments and is recognized as one of the major bacterial pathogens associated with seafood-borne gastroenteritis (Su and Liu, 2007; Letchumanan et al., 2014). Human infection occurs mainly through the consumption of raw or insufficiently cooked seafood, especially products originating from warm coastal waters (Centers for Disease Control and Prevention, 2024). The disease is typically characterized by watery diarrhoea, abdominal cramps, nausea, vomiting, fever, and chills, although severe infections may occur in susceptible individuals (Su and Liu, 2007; Letchumanan et al., 2014; Centers for Disease Control and Prevention, 2024). In the United States, *Vibrio* infections cause approximately 80,000 illnesses annually, with about 52,000 cases associated with contaminated food (Centers for Disease Control and Prevention, 2024). Beyond regional outbreak events, the public-health risk posed by *Vibrio* spp. is expected to increase as rising sea surface temperatures, altered coastal salinity, and globalized seafood trade expand the ecological niche and transmission opportunities of these bacteria (Baker-Austin et al., 2024). In addition to inducing foodborne illnesses in human consumers, *V. parahaemolyticus* poses severe threats to aquaculture. Strains harboring plasmid-encoded pirA/pirB toxin genes are the causative pathogens of acute hepatopancreatic necrosis disease (AHPND), a destructive shrimp disease that triggers enormous economic losses during the early rearing period (Kumar et al., 2020). After being first reported in China in 2009, AHPND was subsequently reported in several shrimp-producing countries and regions, including Vietnam, Thailand, Malaysia, Mexico, and the Philippines (Kumar et al., 2020; González-Gómez et al., 2022). This pathogen thus bridges on-farm disease burdens and postharvest seafood safety risks, highlighting that effective control measures are essential across aquaculture and seafood processing.

Current strategies for controlling *V. parahaemolyticus* in seafood include cold-chain management, thermal treatment, high-pressure processing, irradiation, ultraviolet treatment, chemical sanitizers, organic acids, natural antimicrobials, probiotics, and antibiotics (Liu et al., 2025). These interventions have practical value, but their performance depends strongly on product type, processing conditions, and consumer requirements. Refrigeration can slow bacterial growth but may not eliminate the pathogen; moreover, low-temperature or starvation stress may promote the viable-but-nonculturable (VBNC) state, a survival form first described in *Escherichia coli* and *Vibrio cholerae* in estuarine and marine environments (Li et al., 2014). This physiological state poses great challenges to *Vibrio* control, as VBNC cells can evade routine culture-based assays while remaining metabolically viable. Thermal treatment is effective but unsuitable for raw or ready-to-eat seafood where sensory quality must be preserved. High-pressure processing, irradiation, and ultraviolet treatment can reduce microbial loads, yet they may require specialized equipment, have limited penetration, or affect food structure and quality (Liu et al., 2025). Chemical sanitizers and organic acids can reduce surface contamination, but their effects are often matrix-dependent and may be limited by residue concerns or consumer acceptance. In aquaculture, antibiotics have been used to manage vibriosis; however, the increasing occurrence of antimicrobial-resistant *V. parahaemolyticus* in shrimp and seafood systems has weakened the long-term suitability of antibiotic-based control (Ha et al., 2023; Thaotumpitak et al., 2024). Given these inherent limitations, the development of novel targeted, residue-free, and food-safe antimicrobial interventions is urgently needed.

Lytic bacteriophages have emerged as promising biological agents for controlling foodborne pathogens, given their high specificity toward susceptible bacterial hosts and intracellular replication capability during productive infection (Moye et al., 2018; Vikram et al., 2021). Compared with broad-spectrum chemical or physical decontamination treatments, phage-based biocontrol can selectively target susceptible bacterial pathogens while minimizing effects on non-target food-associated microbiota (Moye et al., 2018). Its application in food production and processing has been explored as both a pre-harvest and post-harvest intervention, particularly for fresh or minimally processed foods where conventional treatments may compromise product quality (Moye et al., 2018; Vikram et al., 2021). To date, most studies on phage applications for controlling *V. parahaemolyticus* have focused on aquaculture, including the isolation and characterization of therapeutic phage candidates as well as prophylactic and therapeutic evaluations in shrimp challenge models (Lomelí-Ortega and Martínez-Díaz, 2014; Jun et al., 2018; Liu et al., 2022; Zhong et al., 2025; Li et al., 2026). In contrast, phage-based interventions aimed at suppressing *V. parahaemolyticus* directly in ready-to-eat food and during the processing stages of seafood production remain largely unexplored (You et al., 2021; Zheng et al., 2025). Accordingly, there is an urgent need to develop phage-mediated post-harvest disinfection strategies that mitigate seafood-derived pathogenic contamination while overcoming the limitations of traditional chemical and physical decontamination treatments.

In this study, we isolated a novel lytic bacteriophage, vB_VpaS_R27Z (R27Z), targeting *V. parahaemolyticus*, and systematically evaluated its morphology, host range, infection dynamics, physicochemical stability, genomic features and biosafety. To assess its application potential in seafood pathogen control, the antibacterial activity and persistence of R27Z were further tested on artificially contaminated fish skin and flesh. This study highlights the great potential of phage R27Z as a novel biocontrol agent for controlling *V. parahaemolyticus* in seafood products.

## 2 Materials and methods

### 2.1 Bacterial strains and growth conditions

The *V*. *parahaemolyticus* ATCC 17802ᵀ (MCCC 1A02609) strain was obtained from the Marine Culture Collection of China (MCCC, https://www.mccc.org.cn/) and used as a target strain for the isolation and propagation of a novel phage. A total of 51 bacterial strains were used to characterize the phage host range in the present study. All strains were deposited in the *Vibrio* Collection Center (VCC, https://www.vibriocollection.com) (Supplementary Table 1). All strains were cultured in rich organic (RO) medium (10 M peptone, 2 M yeast extract, artificial seawater, pH 7.5) at 28°C with shaking at 160 rpm for 6-8 h. Pure cultures were obtained by streak-plating on 1.5% (w/v) agar followed by single-colony selection. Bacterial stocks were preserved in 20% (v/v) glycerol at −20°C and −80°C.

### 2.2 Phage isolation

Environmental water samples were collected from seafood markets in Guangdong Province, China. Samples were filtered through 0.22 μm membrane filters (Millipore, MA, USA) to remove cellular fractions. Phage was enriched by mixing exponential-phase host cultures (OD_600_ = 0.1-0.2) with the filtered samples at a ratio of 1:10 (v/v) and incubating at 28°C with shaking at 150 rpm for 24 h to facilitate phage propagation (Cai et al., 2023; Gao et al., 2024). After incubation, the cultures were centrifuged (8000 × g at 4°C for 15 min), and the supernatants were filtered again through 0.22 μm membranes to obtain cell-free phage lysates. Plaques were obtained using the double-layer agar method (Ding et al., 2025; Kropinski et al., 2009). Serially diluted lysates were mixed with host cells in 0.5% w/v soft agar, overlaid on 1.5% w/v agar plates, and incubated at 28°C for 24 h. Individual plaques were picked and resuspended in SM buffer (50 mM Tris–HCl, 0.1 M NaCl, 8 mM MgSO_4_, 0.1 g/L gelatin; pH 7.5). Purification was performed by at least five successive rounds of plaque isolation (Gao et al., 2024).

### 2.3 Preparation of high-titer phage suspensions

High-titer phage stocks were prepared by a multi-step amplification and purification protocol (Luong et al., 2020; Yamamoto et al., 1970). The purified phage isolate was amplified in exponentially growing host cultures by stepwise scale-up from 20 mL to 1 L. To remove contaminating host nucleic acids, DNase I and RNase A were added to the cell-free lysate at a final concentration of 1 μg/mL and incubated at room temperature for 1 h. NaCl was then added to a final concentration of 1 M, followed by precipitation of phage particles using 10% w/v polyethylene glycol (PEG 8000) at 4°C overnight (Yamamoto et al., 1970). The precipitated phage particles were collected by centrifugation (10,000 × g at 4°C for 60 min) and resuspended in SM buffer. Further purification was performed by cesium chloride (CsCl) density gradient ultracentrifugation (1.3, 1.5, and 1.7 g/cm^3^) (Li et al., 2023) at 200,000 × g at 4°C for 24 h. Intact phage particles formed a distinct band corresponding to their buoyant density (approximately 1.5-1.7 g/cm^3^). The phage-containing fraction was carefully collected and dialyzed against SM buffer using a 30 kDa molecular weight cutoff membrane (Millipore, MA, USA) to remove residual CsCl. Purified phage preparations were stored at 4°C in SM buffer until morphological observation and genomic DNA extraction.

### 2.4 Phage morphological observation

Transmission electron microscopy (TEM) was used to visualize phage morphology. A 10 μL aliquot of the dialyzed, high-titer phage suspension was placed onto a glow-discharged carbon-coated copper grid and allowed to adsorb for 5 min. Excess sample was blotted dry, and the grid was negatively stained with 2% (w/v) phosphotungstic acid (pH 6.5) for 1-2 min. After air-drying, the virion morphology was observed and imaged using a Hitachi HT7700 transmission electron microscope (Hitachi, Japan) at an accelerating voltage of 80 kV (Franken et al., 2020).

### 2.5 One-step growth curve

A one-step growth curve was generated to determine the latent period and burst size of the phage (Anh et al., 2022; Ding et al., 2020; González-Gómez et al., 2022). Exponentially growing *V. parahaemolyticus* ATCC 17802^T^ cultures (OD_600_ ≈ 0.1976; 3.0 × 10^8^ CFU/mL) were infected with the phage lysate at an MOI of 0.77 and incubated for 10 min at room temperature to allow complete adsorption. The mixture was centrifuged (10,000 × g, 4°C, 5 min) and washed twice with RO medium to remove free phages. The bacterial pellet was resuspended in 150 mL of RO medium and incubated at 37°C with shaking. The time immediately after resuspension was defined as t = 0 for the one-step growth experiment. Aliquots were collected every 10 min, serially diluted, and titrated. Burst size was calculated as the ratio of the final maximum phage titer to the initial count of infected bacterial cells (González-Gómez et al., 2022).

### 2.6 Host range analysis

All *Vibrio* strains utilized for host range analysis of phage R27Z are listed in Table 1.

**Table 1:** Host range of phage R27Z.

No. *Vibrio* strains	Lytic ability	No. *Vibrio* strains	Lytic ability
*V. alginolyticus* ATCC 17749^T^	−	*V. parahaemolyticus* ATCC 17802^T^	+
*V. alginolyticus* BVA2	−	*V. parahaemolyticus* vp-HL-202005	−
*V. alginolyticus* JL2674	+	*V. parahaemolyticus* vp-pir-201806	−
*V. alginolyticus* JL3545	+	*V. parahaemolyticus* vp-201911	−
*V. alginolyticus* DA_12	−	*V. parahaemolyticus* D67_T3_16_W6	−
*V. alginolyticus* NB2303_W01	−	*V. parahaemolyticus* DA_HE_3	−
*V. alginolyticus* NB2303_W04	+	*V. parahaemolyticus* DA_HE_4	−
*V. alginolyticus* ATCC 33846	−	*V. parahaemolyticus* VSP1	−
*V. alginolyticus* NB2303_H9B	+	*V. harveyi* BYK0632 (BHV1)	−
*V. alginolyticus* NB2303_H9S	+	*V. harveyi* LMG 4044^T^	−
*V. alginolyticus* NB2303_H8W	−	*V. harveyi* D71_T2_8_W6	−
*V. alginolyticus* JL3565	−	*V. harveyi* JL3577	−
*V. alginolyticus* NB2303_H10	−	*V. harveyi* chan01	−
*V. alginolyticus* JL3557	−	*V. harveyi* ATCC 33842	−
*V. alginolyticus* ZWAG4004 (V.25)	−	*V. owensii* NB2303_I-5	−
*V. alginolyticus* NB2303_I-1Y	−	*V. owensii* NB2303_I-1W	−
*V. alginolyticus* HJ-1	−	*V. owensii* JL2663	−
*V. alginolyticus* NB2303_I-4MI	−	*V. owensii* NB2303_H6	+
*V. alginolyticus* NB2303_H7	−	*V. brasiliensis* NB2303_H2	−
*V. alginolyticus* WPAGA3	−	*V. campbellii* LMG 11216^T^	−
*V. alginolyticus* NB2303_I-8Y	−	*V. campbellii* HJ-2	−
*V. alginolyticus* NB2303_I-7	−	*V. tubiashii* NB2303_W07	+
*V. alginolyticus* NB2303_W09	−	*V. neocaledonicus* VSP3	+
*V. alginolyticus* NB2303_I-4W	−	*V. splendidus* LMG 19031^T^	−
*V. alginolyticus* NB2303_W11	−	*V. anguillarum* LMG 4437^T^	−
		*V. natriegens* WPAGA4	−

(+) representative infection, (−): representative non-infection.

The host range of the phage was evaluated against a diverse panel of 51 distinct *Vibrio* strains. The strain panel consists of 25 *Vibrio alginolyticus*, 8 *V. parahaemolyticus*, 6 *Vibrio harveyi*, 3 *Vibrio owensii*, 2 *Vibrio campbellii*, 2 *Vibrio brasiliensis*, as well as one isolate each of *Vibrio tubiashii*, *Vibrio splendidus*, *Vibrio anguillarum*, *Vibrio neocaledonicus* and *Vibrio natriegens*. Host range analysis was performed according to the routine spot assay (Letarov and Kulikov, 2018; Mirzaei and Nilsson, 2015). Briefly, 5 μL aliquots of the purified phage suspension were titrated onto 1.5% RO agar plates overlaid with 0.5% soft agar containing the respective host strains in the logarithmic phase. Plates were inverted and incubated at 37°C for 7-8 h, and the formation of clear plaques was observed to indicate phage susceptibility (Mirzaei and Nilsson, 2015).

### 2.7 Thermal and pH stability assays

Phage stability under different thermal and pH conditions was evaluated using standard physicochemical stability assays with minor modifications (Liang et al., 2022; You et al., 2021). For thermal stability, phage suspensions (1.5 mL; 1 × 10^7^ PFU/mL) were incubated at 4, 25, 37, 50, 55, and 60°C for 3 h. For pH stability, phage samples were incubated in SM buffer adjusted to pH 2.0-12.0 using HCl or NaOH and maintained at room temperature in the dark for 3 h. Following incubation, residual infectivity was determined by the double-layer agar method. All assays were conducted in triplicate.

### 2.8 Chloroform sensitivity test

To evaluate the chloroform sensitivity of phage, 1 mL of the purified phage lysate (≈10^6^ PFU/mL) was mixed with chloroform at final concentrations of 0%, 2%, and 20% (v/v) (Liang et al., 2022). The mixtures were gently vortexed and incubated in the dark at room temperature for 30 min. Following centrifugation at 5000 × g for 5 min to separate the phases, the upper aqueous layer was collected, and the residual phage titer was quantified using the double-layer plaque assay.

### 2.9 Phage DNA extraction and whole-genome sequencing

Phage genomic DNA was extracted from purified high-titer lysate using a phenol-chloroform method (Sambrook and Russell, 2001). The high-titer phage suspension was treated with proteinase K (0.2 mg/mL), SDS (1% w/v), and EDTA (0.5 M, pH 8.0), followed by incubation at 55°C for 3 h to digest viral proteins and capsid components. The lysate was sequentially extracted with phenol: chloroform: isoamyl alcohol (25:24:1) and chloroform: isoamyl alcohol (24:1), and the aqueous phase was recovered after centrifugation (12,000 × g, 4°C, 10 min). DNA was precipitated with an equal volume of isopropanol at −20°C overnight, centrifuged, washed twice with pre-cooled 70% ethanol, air-dried, and resuspended in 50 μL of TE buffer (10 mM Tris-HCl, 1 mM EDTA, pH 8.0). The DNA concentration and quality were assessed using gel electrophoresis and a NanoDrop spectrophotometer (Thermo Fisher Scientific, MA, USA). Library construction and whole-genome sequencing were performed by Shanghai Hanyu Biotechnology Co. Ltd. (China). The sequencing library was prepared using the NEBNext® UltraTM DNA Library Prep Kit (NEB, USA) and sequenced on the Illumina HiSeq 4000 platform. The complete genome sequence of the phage has been deposited in GenBank under accession number PZ311400.

### 2.10 Bioinformatics analysis of phage genome

Raw sequencing reads were quality-filtered using SOAPnuke (Chen et al., 2018), and the resulting clean reads were assembled de novo with MEGAHIT (Li et al., 2015). The assembled genome was analyzed using PhageTerm (Garneau et al., 2017) on the Galaxy online server (http://galaxy.pasteur.fr) to determine DNA packaging mechanisms and genomic termini. Open reading frames (ORFs) were predicted using Prodigal (Hyatt et al., 2010), and their putative functional annotations were assigned by Basic Local Alignment Search Tool for proteins (BLASTp, https://blast.ncbi.nlm.nih.gov/Blast.cgi?PAGE=Proteins) against the NCBI non-redundant (NR) protein database (E-value ≤10^−5^), with domain-based annotation further refined using InterPro (Blum et al., 2021). To ascertain the biocontrol safety of the phage, the genome was screened for virulence and antibiotic resistance genes using the Virulence Factor Database (VFDB) (Liu et al., 2022) and Comprehensive Antibiotic Resistance Database (CARD) (Alcock et al., 2023), respectively.

For phylogenomic analysis, the complete genome of phage was compared with representative published *Vibrio* phage genomes retrieved from GenBank. The phylogenetic position of phage was inferred using the Genome-BLAST Distance Phylogeny (GBDP) method implemented in the VICTOR platform (Meier-Kolthoff and Göker, 2017). Average amino acid identity (AAI) between the phage and related phages were further calculated using CompareM (version 0.1.2) as an auxiliary measure of proteome-level relatedness. Average Nucleotide Identity (ANI) and intergenomic similarities were computed by VIRIDIC (Moraru et al., 2020), and whole-genome linear comparisons and synteny were visualized using Easyfig (Sullivan et al., 2011).

### 2.11 Bactericidal efficiency assay

To quantitatively evaluate the lytic efficacy of phage, the dynamic growth of the host bacteria was continuously monitored by optical density (OD) using a multifunctional microplate reader (Tecan, Switzerland). Log-phase host cells were dispensed into 96-well culture plates and infected with the phage at MOIs of 0.01, 0.1, 1, 10, and 100. Plates were incubated in the microplate reader, and OD at 600 nm were recorded every 30 min over a 24 h period. Sterile RO medium and phage-free bacterial cultures were established as blank and positive controls, respectively (Zhu et al., 2023).

### 2.12 Antibacterial activity of phage in food

The antibacterial activity of phage R27Z against *V. parahaemolyticus* 17802^T^ in food was evaluated with modifications based on previously reported food-matrix phage application methods (Taj et al., 2024; You et al., 2021). Fresh marine fish specimens were purchased from Nanshan seafood Market, Shenzhen, China (113.92°E, 22.53°N). Following descaling and decapitation, the fish surface was rinsed 2-3 times with sterilized water to remove physical debris and loosely attached surface microbiota. Surface decontamination was then achieved by 75% (v/v) ethanol for 30 min and washed thoroughly with sterile distilled water. The tissues were cut aseptically into approximately 1 cm^3^ pieces and exposed to UV irradiation for 30-60 min. The resulting surface biota-free samples were used for subsequent experimental procedures.

For challenge experiments, fish skin and flesh (muscular tissues) were processed separately. Each sample was inoculated with 100 μL of an exponentially growing *V. parahaemolyticus* ATCC 17802^T^ culture (1 × 10^5^ CFU/mL) for 5 min to allow bacterial attachment on food surface. Phage suspensions were applied at 10^3^ and 10^5^ PFU/mL (100 μL) to achieve MOI 0.01 and MOI 1, respectively. These two MOIs were selected to determine whether R27Z could maintain antibacterial activity in the food matrix at an initial phage dose equal to or substantially lower than the bacterial inoculum. Phosphate-buffered saline (PBS) was used as a negative control group instead of the phage suspension. Samples were collected at 0, 1, 3, 5, and 7 h intervals, homogenized in PBS, and centrifuged (8000 × g, 10 min). The resulting suspensions were subjected to tenfold serial dilutions and enumerated on 1.5% agar plates to calculate colony-forming units (CFUs) and plaque-forming units (PFUs). Changes in viable bacterial counts and recoverable phage titers were quantified in both treated and untreated food-model samples over time. Antibacterial efficacy at each sampling point was expressed either as percentage reduction, calculated as:

$$Phage\;Efficacy\;\left(t_{n}\right)\left(\%\right)=\frac{Nc\;\left(t_{n}\right)-Np\;\left(t_{n}\right)}{Nc\;\left(t_{n}\right)}\times 100$$

Where *Nc* (*t*_*n*_) indicates viable bacterial count in the untreated group at time point, and *Np* (*t*_*n*_), denotes viable bacterial count in phage-treated group at the same time point. Changes in recoverable phage titers over time were used to assess phage persistence and propagation in the seafood matrices.

### 2.13 Statistical analysis

All quantitative experiments were performed in at least three independent biological replicates, and the results are presented as the mean ± standard deviation (SD). Statistical analyses and data visualization were conducted using GraphPad Prism software version 10 (GraphPad Software, San Diego, CA, USA). Statistical significance of data was determined using two-way analysis of variance (ANOVA) followed by Tukey's multiple comparison test. For food models, the significance of variance was analyzed by one-way ANOVA followed by Duncan's multiple comparison. A *p*-value of < 0.05 was considered statistically significant.

## 3 Results

### 3.1 Phage isolation and morphology

In this study, a novel lytic phage R27Z infecting *V. parahaemolyticus* ATCC 17802^T^ was isolated from the sewage drains of Bao'an Agricultural Wholesale Market in Shenzhen, China (113.87°E, 22.59°N). Plaque assays performed on its host *V. parahaemolyticus* ATCC 17802^T^, R27Z produced clear and circular plaques on double-layered agar with a well-defined translucent central zone surrounded by a faint, indistinct peripheral halo (Fig. 1A).

**Fig. 1:**
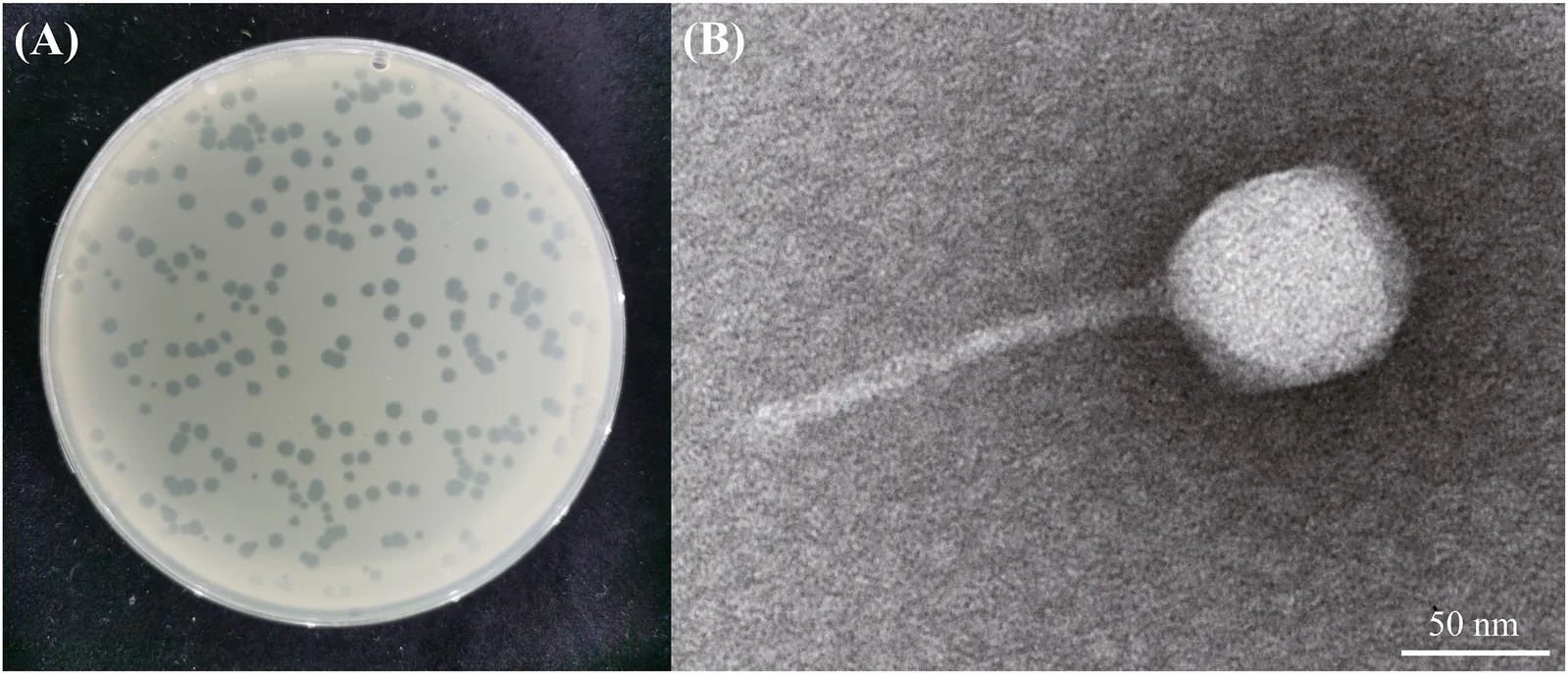
Morphology of phage R27Z. Plaques (A) forming ability on host bacterial lawns and transmission electron microscopic visualization (B) of phage R27Z.

TEM results demonstrated that R27Z exhibits an icosahedral head measuring 84 ± 2 nm in length and 71 ± 2 nm in width, along with a long, flexible, non-contractile tail measuring 141 ± 3 nm (Fig. 1B), consistent with a siphovirus-like morphotype. Following the conventional bacterial virus naming scheme, the isolate was designated vB_VpaS_R27Z, where “vB” denotes bacterial virus, “Vpa” indicates *V*. *parahaemolyticu*s, “S” refers to the siphovirus-like morphotype, and “R27Z” represents the isolate identifier.

### 3.2 Infection dynamics

The infection dynamics of phage R27Z were evaluated using a one-step growth assay using *V*. *parahaemolyticus* ATCC 17802^T^ as the host (Fig. 2). The analysis revealed that phage R27Z exhibited a total lytic cycle of approximately 70 min, comprising a relatively short latent period of about 20 min and a lysis period of roughly 30 min during which the progeny phage titer increased rapidly. This short proliferation cycle, coupled with a burst size of 58 PFU/cell. Together, these results show that R27Z replicated efficiently in its host and produced progeny phages within a relatively short infection cycle under the tested conditions.

**Fig. 2:**
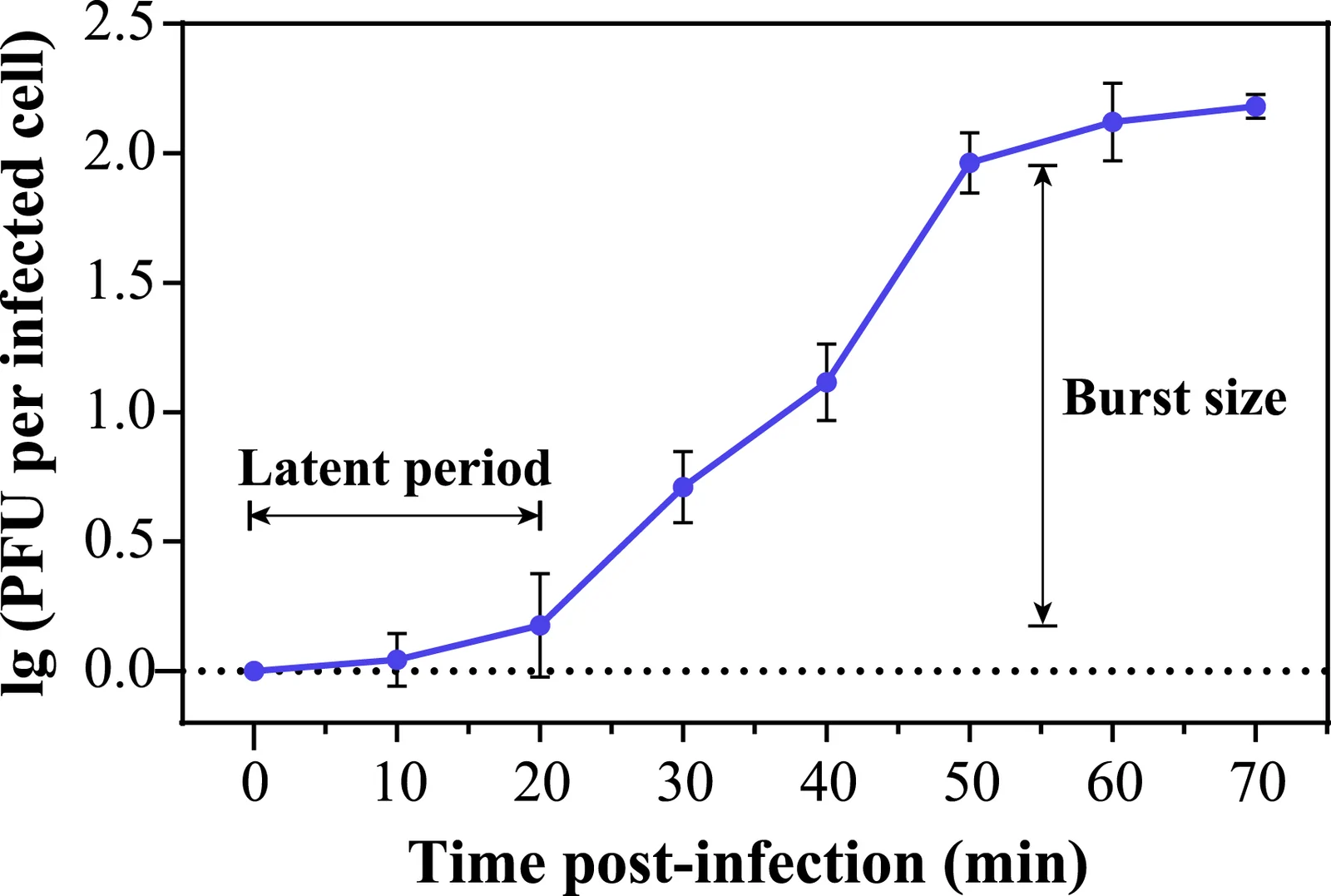
One-step growth curve of phage R27Z. Data points represent the mean ± SD from three independent biological replicates (n = 3). The latent period and burst size are indicated in the figure; the values of phage production are shown as log_10_(PFU per infected cell).

### 3.3 Host lysis spectrum

The host range of phage R27Z was determined using a panel of 51 distinct *Vibrio* strains by spot assay (Table 1). Phage R27Z exhibited a highly specialized and relatively narrow host lytic spectrum and produced clear lysis on 9 strains, including *V. alginolyticus* JL2674, *V. parahaemolyticus* ATCC 17802^T^, *V. neocaledonicus* VSP3, *V. alginolyticus* JL3545, *V. alginolyticus* H9B, *V. tubiashii* NB2303_W07, *V. brasiliensis* NB2303_H6, *V. alginolyticus* NB2303_W04, and *V. alginolyticus* NB2303_H9S.

### 3.4 Resistance to environmental stresses

To evaluate the application potential of phage R27Z in aquaculture environments and food industry, this study thoroughly assessed its survival rates under varying temperature and pH conditions. Thermal stability assays showed that the phage retained high activity across the range of 4°C to 37°C, with no significant changes in survival rates after 3 h of incubation (Fig. 3A). Survival decreased further to approximately 51.69% at 50°C, whereas infectivity declined sharply to 0.09 ± 0.04% at 55°C and was nearly undetectable after 3 h at 60°C. In the pH stability assays, R27Z exhibits optimal stability across a broad pH range of 5.0 to 10.0, with the highest survival observed near neutral pH (Fig. 3B). Extremes outside this optimal range elicited a sharp titer reduction, with no viable particles detected at pH < 4.0 or pH > 11.0. Given that standard aquaculture ecosystems typically operate within a temperature range of 24°C to 30°C and a pH of 7.5 to 8.5, these results indicate that R27Z exhibits short-term tolerance to a range of temperature and pH conditions relevant to potential food and aquaculture applications.

**Fig. 3:**
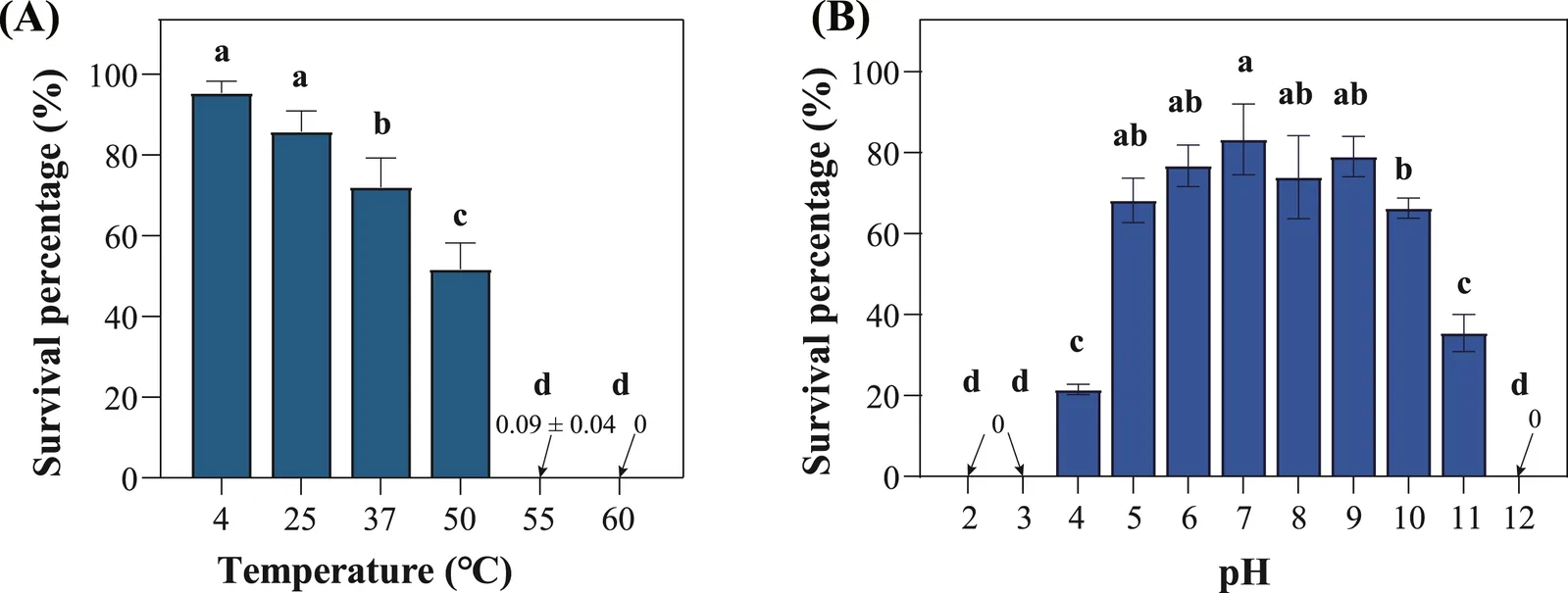
Environmental stability of phage R27Z. (A) Thermal stability of phage R27Z after 3 h incubation at temperatures ranging from 4 to 60°C; (B) pH stability of phage R27Z after 3 h of exposure to pH values ranging from 2.0 to 12.0. Bars represent the mean ± SD (n = 3). Different lowercase letters indicate statistically significant differences among treatments based on one-way ANOVA followed by Tukey's multiple comparison test (*p* < 0.05); identical letters indicate no significant difference.

### 3.5 Chloroform sensitivity

The sensitivity of R27Z to chloroform treatment was evaluated using different chloroform concentrations (Supplementary Fig. 1). Following treatment, no statistically significant differences were observed in phage titer or plaque morphology among groups (*p* > 0.05). Collectively, these results demonstrate a lipid-free capsid in phage R27Z, consistent with the general absence of lipid components in marine tailed bacteriophages.

### 3.6 Genomic characteristics of phage R27Z

Whole-genome sequencing results revealed that phage R27Z possesses a linear double-stranded DNA genome of 51,052 bp with an average GC content of 41.65% (Fig. 4). A total of 64 open reading frames (ORFs) and two tRNA genes (*tRN*A^*Ile*^ and *tRNA*^*Ser*^) were predicted in the genome. Based on sequence similarity and domain annotation, putative functions were assigned to the predicted ORFs, which were classified into five functional modules: DNA metabolism and replication (8 ORFs), structural proteins (8 ORFs), DNA packaging (3 ORFs), and host lysis (1 ORF), and other functions (8 ORFs); the remaining ORFs were designated as encoding hypothetical proteins. The DNA metabolism and replication module encodes a DNA polymerase (ORF33), a DNA primase/helicase (ORF35), ribonucleotide reductases (ORF27 and 28), and thymidylate synthase (ORF40). Genes predicted to encode major head protein (ORF10), scaffolding protein (ORF9), and tail fiber proteins (ORF15 and 17) were grouped within the structural module. Genome encapsidation is directed by DNA packaging module containing a terminase small subunit (ORF3), a terminase large subunit (ORF6), and a portal protein (ORF7). The host lysis module is anchored by a putative endolysin (ORF4), which is identified adjacent to a putative membrane lipoprotein (ORF1), and further conserved-domain analysis identified a lysozyme-like domain consistent with a predicted peptidoglycan-hydrolase function. ORF1 showed sequence similarity to annotated unimolecular spanins and was predicted to contain an N-terminal Sec/SPII lipoprotein signal peptide and a C-terminal transmembrane helix, a topology consistent with a putative unimolecular spanin. Additionally, auxiliary metabolic genes (AMGs) were identified within the genome, encompassing phosphoribosyl-ATP diphosphatase (ORF44), ATP-grasp enzyme (ORF48), and carbamoyl phosphate synthetase (ORF54). No antibiotic resistance genes, virulence factors, or lysogeny-associated sequences were detected upon screening against the CARD and VFDB databases, supporting the suitability of phage R27Z for biocontrol applications.

**Fig. 4:**
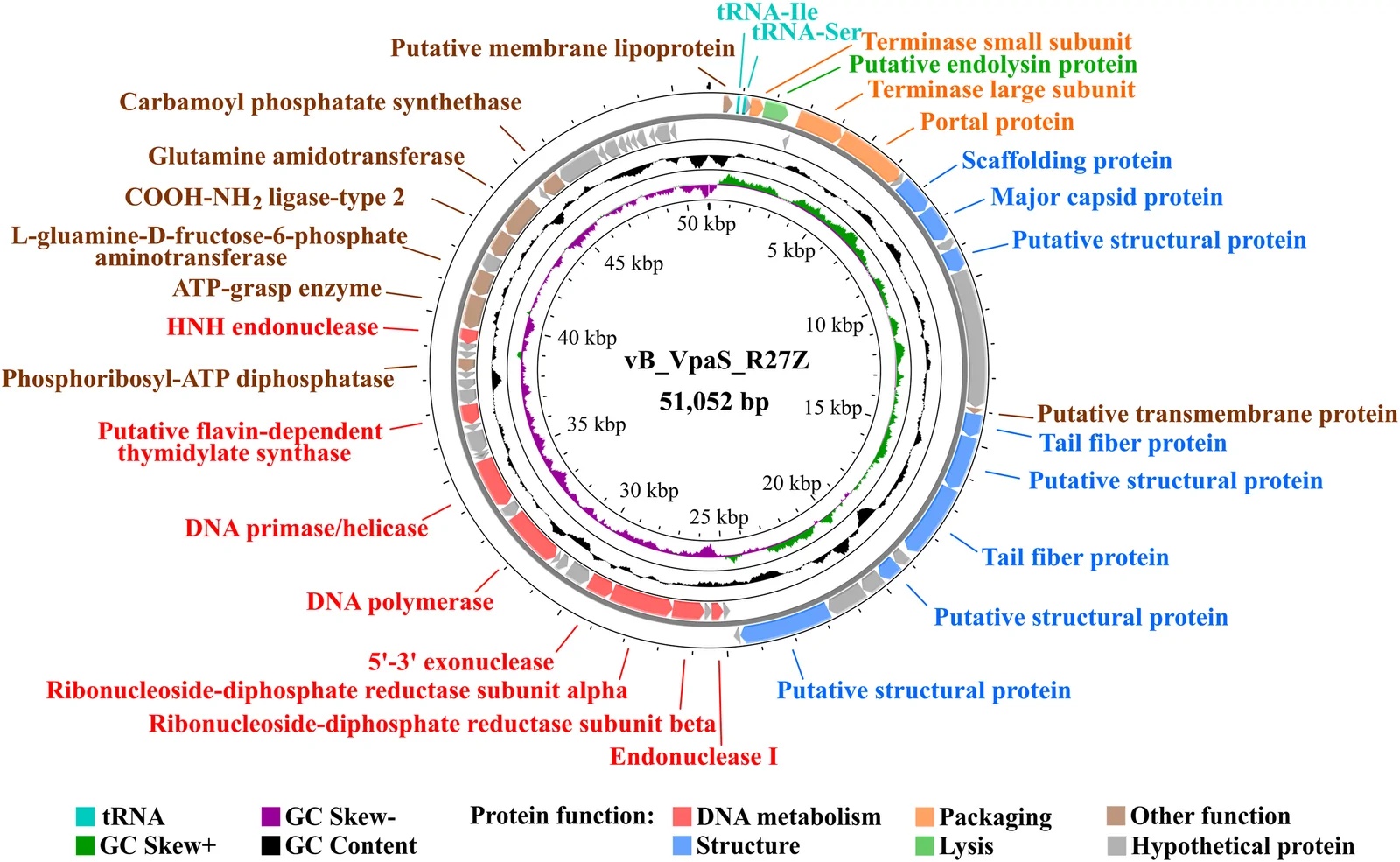
Genomic map of phage R27Z. Predicted coding sequences (CDSs) are shown on the outer ring and color-coded according to their putative functions. Clockwise and counterclockwise arrows indicate the forward and reverse orientations of the predicted CDSs, respectively. The positions of the two predicted tRNA genes are also indicated. The inner rings represent GC content and GC skew, and the genome scale is shown in kbp.

### 3.7 Phylogenetic and comparative genomics analysis of R27Z

R27Z shared high nucleotide similarity with several previously reported *Vibrio* phages including KIT05, vB_ValP_IME234, vB_VpP_BA6, vB_ValP_IME271, VpJYP1, vB_ValC_WD615, and VPp1. The highest sequence similarities were observed with Vibriophage KIT05 (95.90% identity, 95% query coverage) and vB_ValP_IME234 (96.64% identity, 87% query coverage) (Table 2). Although these phages exhibited high genomic similarity, they were isolated from different hosts and environmental sources. KIT05, VPp1, and vB_VpP_BA6 were described as *V. parahaemolyticus*-infecting phages, whereas vB_ValP_IME271 and vB_ValC_WD615 were described as *V. alginolyticus*-infecting phages; vB_ValP_IME234 was reported to infect both *V. alginolyticus* and *V. parahaemolyticus*. And they were isolated from diverse environments such as marine sediments, shellfish, and seafood market sewage in different geographical regions (Anh et al., 2022; Li et al., 2018, 2019, 2021; F. Li et al., 2019; Yang et al., 2019; Dai et al., 2024).

**Table 2:** The ANI and AAI between vB_VpaS_R27Z and related phages.

Scientific name	Accession	Query cover (%)	ANI (%)	Genes	Mean AAI	Orthologous genes radio (%)
KIT05	AP019417.1	95	95.9	64	95.56	95.31
vB_ValP_IME234	MW748992.2	87	96.64	66	93.53	90.62
vB_VpP_BA6	MK483107.1	89	92.09	63	92.8	92.06
vB_ValP_IME271	MF431726.1	90	93.29	65	95.05	89.06
VpJYP1	MW217515.1	88	97.2	66	92.64	87.5
vB_ValC_WD615	OR908438.1	84	97.52	69	92.97	85.94
VPp1	KJ936628.1	84	96.21	65	90.88	85.94

Phylogenetic analysis based on the GBDP method placed R27Z within a well-supported clade containing KIT05, vB_ValP_IME271, vB_ValP_IME234, vB_VpP_BA6, VPp1, vB_ValC_WD615, and VpJYP1 (Fig. 5A). Within this clade, R27Z clustered most closely with KIT05, which was consistent with the high sequence similarity revealed by BLASTn analysis. These related phages also showed comparable genome size (50,336–51,052 bp) and GC content (41.0-42.0%), supporting their close evolutionary relationship.

**Fig. 5:**
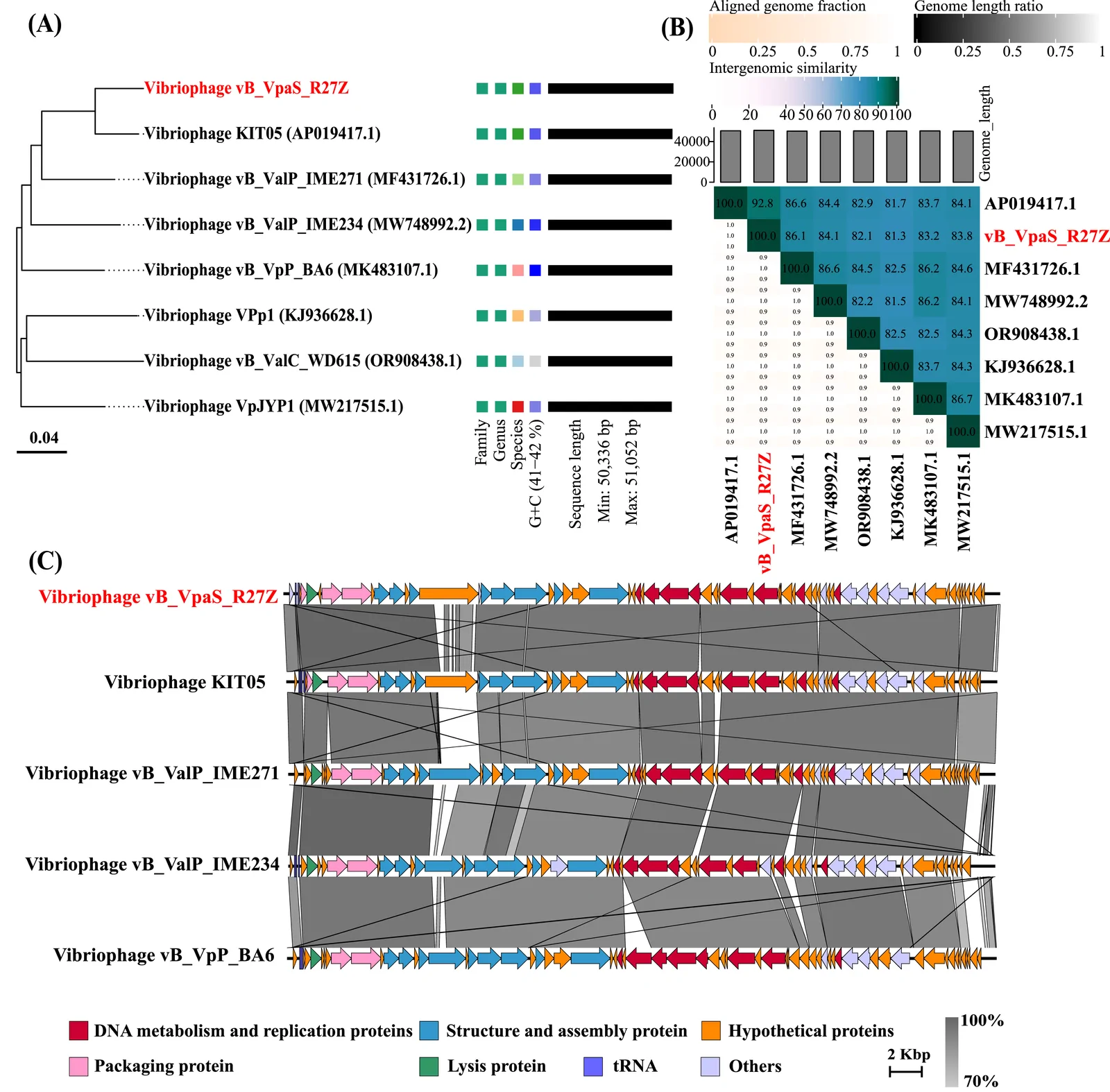
Phylogenetic and comparative genomics analysis of phage R27Z. (A) Phylogenetic tree, including R27Z and seven closely related phages. (B) Heatmap between R27Z and seven closely related phages generated by VIRIDIC incorporating pairwise intergenomic similarities values (right half) and alignment indicators (left half and top annotation). (C) Whole-genome comparative analysis of R27Z with four closely related *Vibrio* phages. The genomes are aligned linearly to visualize homologous regions and structural conservation. Arrows represent predicted coding sequences and their transcriptional orientation, color-coded according to their putative functions. Gray connecting regions indicate sequence similarity between genomes, with darker shading representing higher similarity. R27Z is highlighted in red.

Intergenomic similarity analysis using VIRIDIC further indicated that R27Z shared 81.3% to 92.8% similarity with its seven related phages, with the highest value (92.8%) shared with KIT05 (Fig. 5B). These results support the placement of R27Z within the same genus-level lineage as these phages. While according to the ICTV criteria, the ANI threshold for species definition is 95%. Consequently, R27Z is recognized as a novel species within this genus. The AAI analysis provided additional support for this relationship (Table 1). R27Z shares the highest proportion of orthologous genes (95.31%) and the highest average amino acid identity (95.56%) with KIT05.

Whole-genome linear comparison of R27Z with four phylogenetically closest phages (KIT05, vB_ValP_IME271, vB_ValP_IME234, and vB_VpP_BA6) revealed a high degree of genomic synteny (Fig. 5C). The genomic arrangement of functional modules was largely conserved across all compared phages, particularly in regions corresponding to DNA metabolism and replication, structural assembly, DNA packaging, and host lysis, which maintain consistent relative positions and transcription orientations across genomes. At the same time, localized sequence divergence was apparent at several loci, particularly in regions enriched in hypothetical proteins and at positions near the genome termini. Together, these results indicate that R27Z shares a conserved genomic backbone with related vibriophages, while retaining sufficient sequence divergence to support its designation as a distinct species.

### 3.8 Phage bactericidal efficiency in vitro

The bactericidal efficacy of phage R27Z against *V. parahaemolyticus* ATCC 17802^T^ was further evaluated by monitoring bacterial growth kinetics at different MOIs over 24 h (Fig. 6). The phage-free control culture exhibited a typical exponential phase and entered into a stationary plateau at approximately 8-10 h. In contrast, host cultures inoculated with R27Z resulted in a clear suppression of bacterial growth across all tested MOIs (0.01, 0.1, 1, 10, and 100). Higher MOIs generally produced stronger inhibition, particularly during the exponential phase. The maximum lytic differential between the control and treated cultures was observed between 3 and 7 h of co-incubation, during which OD_600_ values in the phage-treated groups remained substantially lower than those of the untreated control.

**Fig. 6:**
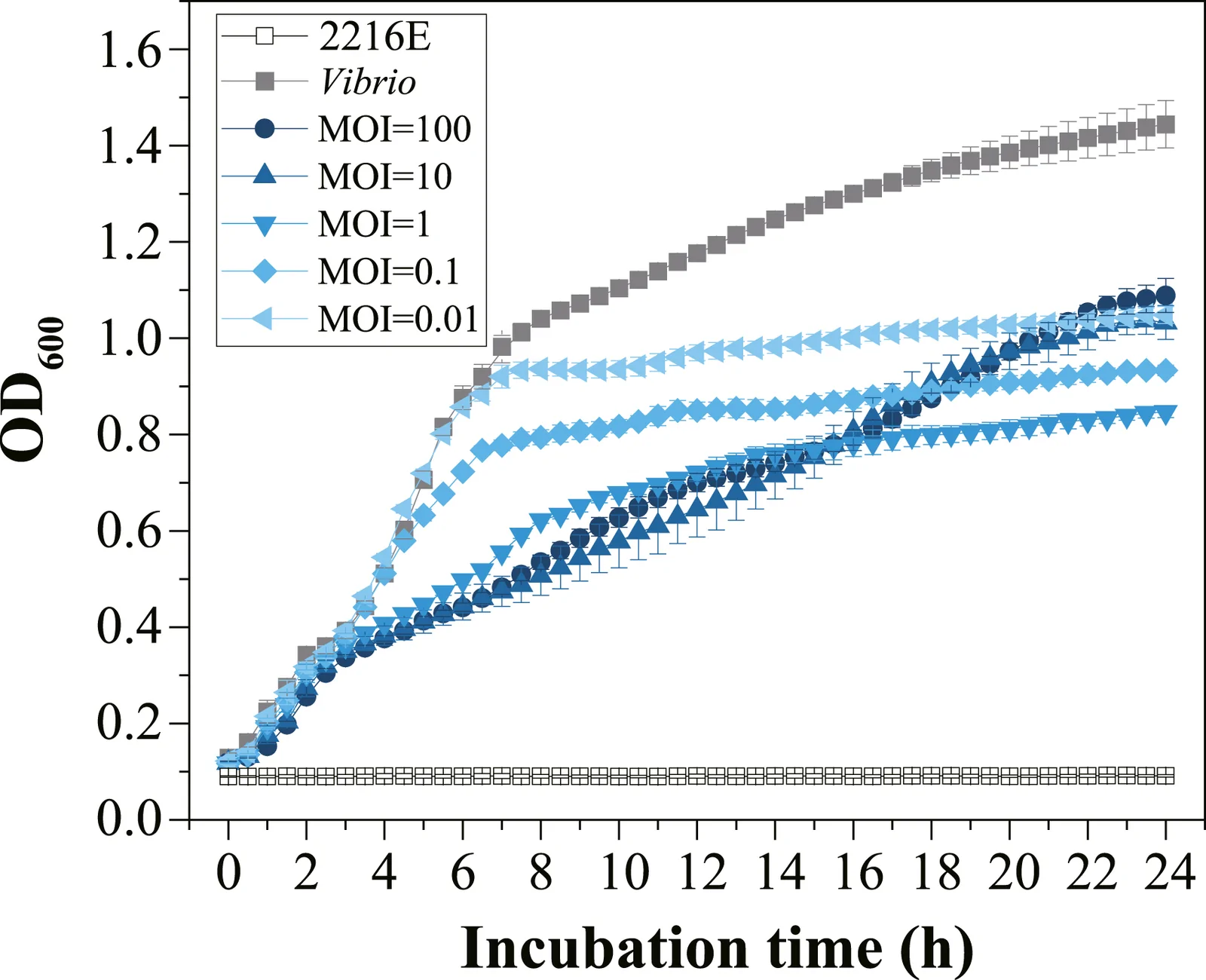
In vitro inhibition of *V. parahaemolyticus* by p**hage R27Z.***V. parahaemolyticus* ATCC 17802ᵀ was treated with R27Z at MOIs of 0.01, 0.1, 1, 10, and 100, and bacterial growth was monitored by OD_600_ over 24 h. Data are shown as the mean ± SD of three independent experiments.

### 3.9 Antibacterial activity of phage R27Z in seafood surfaces

The antibacterial activity of phage R27Z was evaluated on artificially contaminated fish muscle and fish skin surfaces using *V. parahaemolyticus* ATCC 17802^T^ as the target host (Fig. 7). Under the tested conditions, phage treatment significantly suppressed bacterial proliferation on both food models after 7 h of incubation at 28°C (*p* < 0.05). On fish muscle, treatments at MOIs of 1 and 0.01 significantly mitigated bacterial proliferation, resulting in viable cell reductions of 3.62 log_10_ CFU/cm^3^ and 3.26 log_10_ CFU/cm^3^ (Fig. 7A), respectively, compared to the untreated groups, where bacterial load increased by approximately 3.32 log_10_ CFU/sample over the same period. These results correspond to 99.97% and 99.94% bacterial inactivation, indicating robust antibacterial potential within 7 h. Similarly, on fish skin, the cell count reduced to 2.84 log_10_ CFU/cm^2^ (99.85%) and 2.24 log_10_ CFU/cm^2^ (99.42%) at MOI 1 and MOI 0.01, respectively (Fig. 7C). In both food models, the higher phage input generally produced greater bacterial reduction. The consistently lower reduction magnitude on skin compared to muscle likely reflects the physical constraints imposed by the skin matrix.

**Fig. 7:**
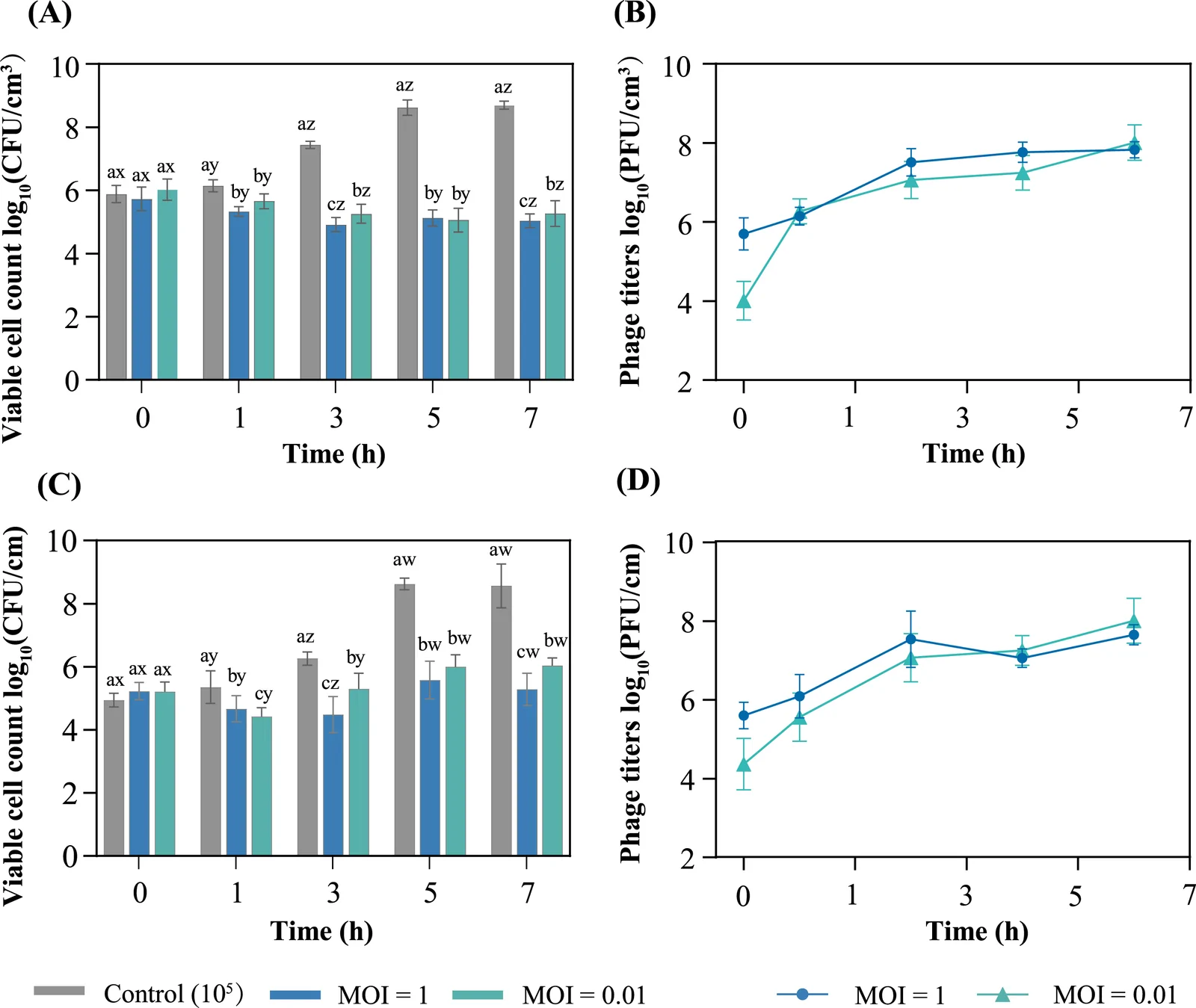
Phage R27Z inhibition efficacy and replication dynamics on seafood surfaces. (A and B) Viable bacterial counts and recoverable phage titers on fish muscle. (C and D) Viable bacterial counts and recoverable phage titers on fish skin. Samples were incubated at 28°C and analyzed over 7 h. Each bar and dot represent the mean of three replications (n = 3), and the SD is presented with error bars in the graphs. Lowercase letters (a-c) indicate a significant difference (*p* < 0.05) among treatments at the same time points, while x-z indicate differences across incubation time points within the same treatments, respectively. Values sharing the same letters are not significantly different.

Furthermore, recoverable phage titers also increased over time in both food systems (Fig. 7B–D). The average antimicrobial performance of phage R27Z was enhanced to 35.5%, and 81.90% yielded in 105 and 190-fold increases in phage titers at MOI 1 and MOI 0.01, respectively. Recovered phage titers ranged from 5.6 × 10^7^ PFU/cm^2^ to 1.1 × 10^8^ PFU/cm^2^, confirming sustainable phage propagation and activity. These results indicate that R27Z remained active on both seafood surfaces and retained the capacity to replicate in the presence of its host under the experimental conditions.

## 4 Discussion

*V. parahaemolyticus* is the dominant pathogen driving foodborne illnesses in numerous countries and coastal regions. Its mitigation poses distinct challenges for raw and minimally processed seafood, as antimicrobial treatments need to lower pathogen loads without markedly compromising product quality (Su and Liu, 2007; Letchumanan et al., 2014). In the study, a novel lytic phage R27Z was characterized at the morphological, biological, and genomic levels and further evaluated on artificially contaminated fish muscle and fish skin. The primary novelty of this work resides in expanding phage efficacy evaluation from in vitro cultures to representative seafood matrices, providing experimental evidence for the postharvest biocontrol potential of R27Z.

R27Z exhibited a siphovirus-like morphology, with an icosahedral head and a long non-contractile tail. It lysed 9 of 51 tested *Vibrio* strains, corresponding to a strain-level host coverage of 17.65%. Host recognition by tailed phages is generally mediated by receptor-binding proteins that interact with bacterial surface structures such as lipopolysaccharides, pili, flagella, capsules, or outer-membrane proteins (Nobrega et al., 2018). Two putative tail fiber proteins, ORF15 and 17, were identified in R27Z genome and may contribute to adsorption, although their receptor specificity remains unknown. The restricted host range of R27Z limits its use as a standalone biocontrol agent in industrial seafood applications, where heterogeneous *V. parahaemolyticus* populations may not all be susceptible to a single phage (Hyman, 2019). Similar strain-level specificity has been reported for other *V. parahaemolyticus* phages, and phage combinations with complementary host ranges have shown greater bacterial suppression than individual phages (Tan et al., 2021). Therefore, R27Z may be more appropriately considered as a candidate component of a phage cocktail, in which complementary phages could broaden strain coverage and improve the robustness of pathogen control (Fong et al., 2021; Tan et al., 2021).

Comparison with the closely related phage KIT05 revealed an apparent discordance between genomic conservation and virion morphology. Although KIT05 has a genome similar in size to that of R27Z, it was reported to possess a short, non-contractile tail of approximately 24.1 nm, whereas R27Z tail measured approximately 141 nm (Anh et al., 2022). In long-tailed phages, tail length is closely associated with the tail morphogenesis machinery, particularly the tape-measure protein, whereas distal tail fibers are primarily involved in host recognition (Linares et al., 2020). We further performed a multi-step genomic screening workflow combining PhageTailFinder, coiled-coil prediction, and HHpred remote-homology search to identify putative TMP. Nevertheless, no high-confidence TMP candidate could be retrieved for R27Z The canonical Pfam profile PF01551, which is widely used for siphoviral TMP identification, frequently fails to capture highly divergent TMP sequences, including many myoviral homologs. Notably, as a reported podovirus, KIT05 does not employ a TMP-dependent molecular-ruler system for tail assembly; its short tail is formed by a compact stacked portal-adaptor-nozzle-needle protein complex. Collectively, this pattern supports a scenario of modular replacement of the tail-morphogenesis cassette, in which most of the genomic backbone remains conserved while exchange of tail-structural genes drives large morphological divergence. HHpred analysis of ORF17 identified a high-confidence match to the probable central straight fiber of the long-tailed bacteriophage T5 (PDB 7ZQB; probability, 99.72%; E-value, 7.9 × 10^−17^; 186 aligned columns), providing an additional, albeit indirect, line of evidence consistent with the long-tailed morphology of R27Z (Linares et al., 2023). Comparable morphology–genome incongruence has been reported for other phages. For example, *V. parahaemolyticus* phage vB_VpS_PG28 displays a contractile, myovirus-like tail despite clustering with siphovirus-like phages in terminase- and DNA polymerase-based phylogenies (Tian et al., 2022). Morphological variation has also been documented among genomically related *Kayfunavirus* phages. vECPPW10 and JSS1 possess short or stubby non-contractile tails, whereas CY1 was reported to have a long tail of approximately 180 nm (Cao et al., 2023; Wintachai et al., 2026). Pairwise BLASTn comparisons further showed substantial nucleotide similarity between CY1 and the short-tailed phages examined in the present comparison. These observations indicate that substantial genomic similarity does not necessarily translate into identical tail morphology. The available sequence data therefore do not provide a definitive molecular explanation for the marked difference in reported tail length. Importantly, Multiple independent TEM experiments were repeated (Supplementary Fig. 2.), confirming that the morphological features of phage R27Z are consistent with those described in the prior manuscript version. The discrepancy may partly result from methodological variation during TEM preparation, or reflect localized divergence in uncharacterized tail-assembly determinants despite conservation of the broader genomic backbone, consistent with the mosaic nature of phage genome evolution (Williamson et al., 2012; Dion et al., 2020). Direct comparison of R27Z and KIT05 under identical experimental conditions, together with further structural and functional characterization of candidate tail-morphogenesis proteins, would be required to distinguish between these possibilities.

Following successful infection, the efficient propagation of R27Z is driven by a highly autonomous replication machinery. The phage exhibits a rapid lytic cycle, characterized by a short latent period (20 min) and a moderate burst size (58 PFU/cell). KIT05, in comparison, was reported to have a latent period of approximately 40 min and a burst size of 18 PFU per infected cell (Anh et al., 2022). These values indicate that R27Z completed its lytic cycle more rapidly and released more progeny under the experimental conditions used in the present study. Rather than maximizing progeny output per cell, this replication dynamic reflects an evolutionary trade-off that favors rapid host cell lysis, allowing the phage to exponentially outpace bacterial proliferation during the critical early stages of contamination (Abedon et al., 2003). This efficiency is underpinned by phage-encoded replication enzymes, including DNA polymerase and DNA primase/helicase, which reduce the dependence on host DNA synthesis mechanisms. Two tRNAs, tRNA-Ile and tRNA-Ser, were also identified; whether they improve translational efficiency requires codon-usage and expression analysis (Bailly-Bechet et al., 2007; Dion et al., 2020). In addition to replication-related genes, R27Z contains a putative host-lysis module involving ORF4 and ORF1. To further assess their possible roles in phage-mediated lysis, ORF4 and ORF1 were subjected to additional sequence-similarity, conserved-domain, signal-peptide, and membrane-topology analyses, using InterPro, NCBI CD-Search, SignalP 6.0, and DeepTMHMM. ORF4 was predicted to encode an endolysin containing a lysozyme-like domain and is non-contiguously inserted between the terminase small subunit (ORF3) and large subunit (ORF6), a similar genomic arrangement also observed in phage KIT05 and VPp1 (Anh et al., 2022; Li et al., 2018). Phage endolysins are peptidoglycan hydrolases that degrade the bacterial cell wall during the terminal stage of infection, whereas disruption of the outer membrane of Gram-negative hosts generally requires additional lysis functions, including spanins (Cahill and Young, 2019). ORF1 was predicted to contain an N-terminal Sec/SPII lipoprotein signal peptide and a C-terminal transmembrane helix, an architecture consistent with a putative unimolecular spanin (Kongari et al., 2018). However, the predicted roles of ORF1 and ORF4 remain to be experimentally validated. Recombinant endolysins derived from the *V. parahaemolyticus* phages qdvp001 and VPp1 have demonstrated lytic activity against *V. parahaemolyticus*, with LysVPp1 showing enhanced hydrolytic activity against EDTA-permeabilized cells (Wang et al., 2016; Li et al., 2018). These findings highlight the potential of vibriophage endolysins as independently applied antimicrobials, but the outer membrane of Gram-negative bacteria remains an important barrier to externally applied endolysins (Sisson et al., 2024). Functional characterization of recombinant ORF4, including assays using intact and outer-membrane-permeabilized cells, will therefore be required to determine its enzymatic activity, antibacterial spectrum, and potential as a standalone antimicrobial.

Currently, *Vibrio* contamination of aquatic foods remains an important food-safety concern, and protein-rich seafood matrices can provide nutrients for microbial proliferation during inadequate handling (Su and Liu, 2007; Letchumanan et al., 2014). In the present study, R27Z demonstrated effective reduction of *V. parahaemolyticus* in fish muscle and skin matrices, indicating its potential as a postharvest biocontrol intervention. During the 7-h challenge period, R27Z significantly reduced *V. parahaemolyticus* populations at both MOIs of 1 and 0.01 (*p* < 0.05), accompanied by an increase in recoverable phage titers, suggesting active replication in the inoculated host population under the tested conditions. The marked increase of bacterial numbers in untreated controls was expected because the experimental design was intended to evaluate phage-mediated suppression under host growth-permissive conditions rather than to reproduce refrigerated seafood storage. Rapid growth of *V. parahaemolyticus* at warm temperatures has previously been documented in seafood matrices; for example, a specific growth rate of 0.558 log CFU/h was reported in salmon meat at 30°C, while a predictive model for raw marinated crab estimated a 1-log increase within 4.7 h at 28°C (Yang et al., 2009; Chung et al., 2019). In the present study, exponentially growing bacterial cells were introduced into fish matrices and incubated at 28°C, allowing evaluation of the maximal antibacterial potential of R27Z when *V. parahaemolyticus* is metabolically active. The reduction achieved by R27Z was comparable to or greater than those reported for several previously characterized *V. parahaemolyticus* phages. For example, phages vB_Va_ZWPVA056 and OY1 achieved reductions of approximately 1.14–2.42 log CFU/mL under food-related conditions, although these studies used different matrices, phage doses, and incubation periods (Gao et al., 2022; Xu et al., 2025). The stronger reduction observed in the present study may be attributed to the efficient replication characteristics of R27Z and the growth-permissive conditions used for challenge evaluation. The slightly lower antibacterial effect observed on fish skin compared with fish muscle may result from matrix-specific limitations, including heterogeneous bacterial attachment, reduced phage accessibility, and spatial constraints affecting phage-host encounters on complex food surfaces (Moye et al., 2018).

The fish matrix assay was conducted using surface-decontaminated tissues to minimize interference from indigenous microorganisms and enable quantitative evaluation of R27Z activity against the target pathogen. Similar ethanol/UV decontamination procedures have previously been applied in controlled phage challenge assays using fish matrices (Ha et al., 2023). However, ethanol/UV pretreatment may alter the physicochemical properties of fish tissues, as ethanol can affect the structural and aggregation behavior of fish myofibrillar proteins and UV treatment can modify the physicochemical characteristics of fish fillets (Monteiro et al., 2019; Xiong et al., 2025). Because matrix properties and surface structure can influence phage accessibility and phage–bacterium contact in solid foods, such pretreatment could potentially affect phage–host interactions, although this was not directly assessed in the present study (Guenther et al., 2009; Shannon et al., 2020). Moreover, removal of the indigenous microbiota means that this model does not fully reproduce the microbial complexity of untreated seafood. Native microbiota may influence phage efficacy by altering bacterial physiological states, competing for ecological niches, or modifying phage–host interactions within food matrices (Denes and Wiedmann, 2014; Shannon et al., 2020). Therefore, the observed antibacterial activity should be interpreted within the context of a controlled, surface-decontaminated challenge model, and future studies should validate R27Z in naturally contaminated seafood containing native microbiota.

The absence of bacterial regrowth during the 7-h treatment period indicates sustained short-term suppression; however, this observation should not be interpreted as evidence of long-term preservation capability. The purpose of the present model was to assess R27Z as a postharvest intervention capable of rapidly reducing *V. parahaemolyticus* contamination, rather than as a replacement for refrigerated storage. In practical seafood production chains, contamination events may occur during processing, handling, and distribution, and temperature fluctuations or interruptions in cold-chain management may provide opportunities for pathogen persistence and proliferation (Ndraha et al., 2022; Brauge et al., 2024). Accordingly, R27Z may be particularly valuable as a complementary control measure applied before refrigeration or during handling stages where rapid pathogen reduction is required. Nevertheless, several *V. parahaemolyticus* phages have demonstrated activity under refrigerated conditions, suggesting that low-temperature biocontrol represents a possible additional application strategy. For example, VPT02 reduced *V. parahaemolyticus* contamination on raw fish slices at 5°C, while pVp-JHP1101 maintained antibacterial activity against *V. parahaemolyticus* in clams during 72 h of storage at 4°C (You et al., 2021; Park et al., 2025). Whether R27Z can provide comparable protection during extended chilled storage remains to be investigated.

Beyond antibacterial efficacy, the practical application of R27Z as a food biocontrol agent depends on its stability under processing-related conditions and its safety profile. R27Z remained active across a broad temperature range (4–50°C) and pH range (5.0–10.0) after 3 h exposure, suggesting that it can tolerate physicochemical variations encountered during seafood handling. However, this stability should not be interpreted as evidence for prolonged antibacterial activity during refrigerated storage. Refrigeration primarily limits the growth of *V. parahaemolyticus* rather than promoting continuous phage replication, whereas contamination events during processing, handling, or temperature-abuse conditions may provide opportunities for pathogen persistence. Therefore, the observed stability of R27Z is more relevant to its compatibility with postharvest intervention procedures than to long-term preservation during chilled storage (Ndraha et al., 2022; Brauge et al., 2024). Future studies should evaluate R27Z under realistic cold-chain conditions to determine whether it can contribute to extended seafood protection. Safety evaluation of phage applications in food systems requires consideration of both genomic characteristics and potential consequences of bacterial lysis. Whole-genome analysis revealed that R27Z lacks identifiable genes associated with virulence, antimicrobial resistance, or lysogenic functions, supporting its suitability as a lytic biocontrol candidate (Moye et al., 2018). Nevertheless, because *V. parahaemolyticus* is a Gram-negative bacterium, phage-mediated lysis may release lipopolysaccharide-containing outer membrane components, which should be considered in future biosafety assessments (Dufour et al., 2017; Liu et al., 2021). Beyond microbiological safety, previous food phage applications have generally reported no detectable adverse effects on sensory attributes; however, the effects of R27Z treatment on seafood appearance, texture, and odor were not evaluated in the present study and should be determined before practical application (Perera et al., 2015). Collectively, these results support R27Z as a promising candidate for targeted postharvest control of *V. parahaemolyticus*, while further validation in complex seafood systems is required.

## 5 Conclusion

R27Z is a lytic *V*. *parahaemolyticus* phage with a defined host range, rapid infection kinetics, and good stability within the tested temperature (4-50°C) and pH ranges (pH 5.0-10.0). Genome annotation did not identify virulence, antibiotic-resistance, or lysogeny-related genes. R27Z also reduced *V. parahaemolyticus* on fish tissue surfaces under the tested conditions, and recoverable phage titers remained increased during the experimental period. These findings support the further evaluation of R27Z as a candidate for seafood-related biocontrol. Future work should test its performance under storage-relevant conditions, assess its activity in more complex food matrices, and examine its use in multi-phage formulations.

## CRediT authorship contribution statement

**Hanxi Xiao:** Investigation, Visualization, Methodology, Formal analysis, Writing – original draft. **Muhammad Imran Taj:** Investigation, Formal analysis. **Biao Zhu:** Investigation, Formal analysis. **Chaoyin Xia:** Investigation, Formal analysis. **Mingfu Wang:** Writing – review & editing. **Bu Xu:** Conceptualization, Writing – review & editing. **Rui Zhang:** Conceptualization, Writing – review & editing. All authors read and approved the final version of the manuscript.

## Data availability

The complete genome sequence of Vibriophage vB_VpaS_R27Z generated in this study has been deposited in the NCBI GenBank database under accession number PZ311400.

## Funding

This work was supported by the 10.13039/501100004791Shenzhen 10.13039/100012335Medical Research Fund (B2503003), the 10.13039/501100001809National Natural Science Foundation of China (32570002), the 10.13039/501100004791Shenzhen Science and Technology Program (JCYJ20241202124403006), the 10.13039/501100021171Guangdong Basic and Applied Basic Research Foundation (2025A1515010903) and the 10.13039/501100002858China Postdoctoral Science Foundation (2024M762131).

## Declaration of competing interest

The authors declare the following financial interests/personal relationships which may be considered as potential competing interests:

Hanxi Xiao, Bu Xu, Rui Zhang, Biao Zhu, and Chaoyin Xia are named inventors on a pending Chinese patent application (2025119855724) entitled “Isolation and application of a lytic *Vibrio parahaemolyticus* bacteriophage vB_VpaS_R27Z″, which is related to the work reported in this manuscript. The remaining authors declare no competing financial interests or personal relationships that could have influenced the work reported in this paper.

## Appendix A Supplementary data

Fig. S1 Chloroform sensitivity assay of phage R27Z at different chloroform concentrations. Phage titers were determined by the double-layer agar method and expressed as mean ± SD (n = 3). Different letters indicate statistically significant differences (P < 0.05), whereas identical letters indicate no significant difference. Chloroform concentrations: 0%, 2%, and 20%. Multimedia component 1

Fig. S2 Representative transmission electron microscopy (TEM) images of phage R27Z from multiple independent observations. R27Z consistently exhibited an icosahedral head and a long, non-contractile tail. Scale bars are indicated in each image.Multimedia component 2
