# Supplementary material for: Isolation, characterization, and biocontrol potential of a novel lytic phage vB_VpaS_R27Z against Vibrio parahaemolyticus for mitigating seafood contamination
Source: Curr Res Food Sci. 2026 Oct 22;13:101602. doi: 10.1016/j.crfs.2026.101602 (PMC13737724; doi:10.1016/j.crfs.2026.101602)
Supplement: Supplementary file 1 [file mmc1.pdf]

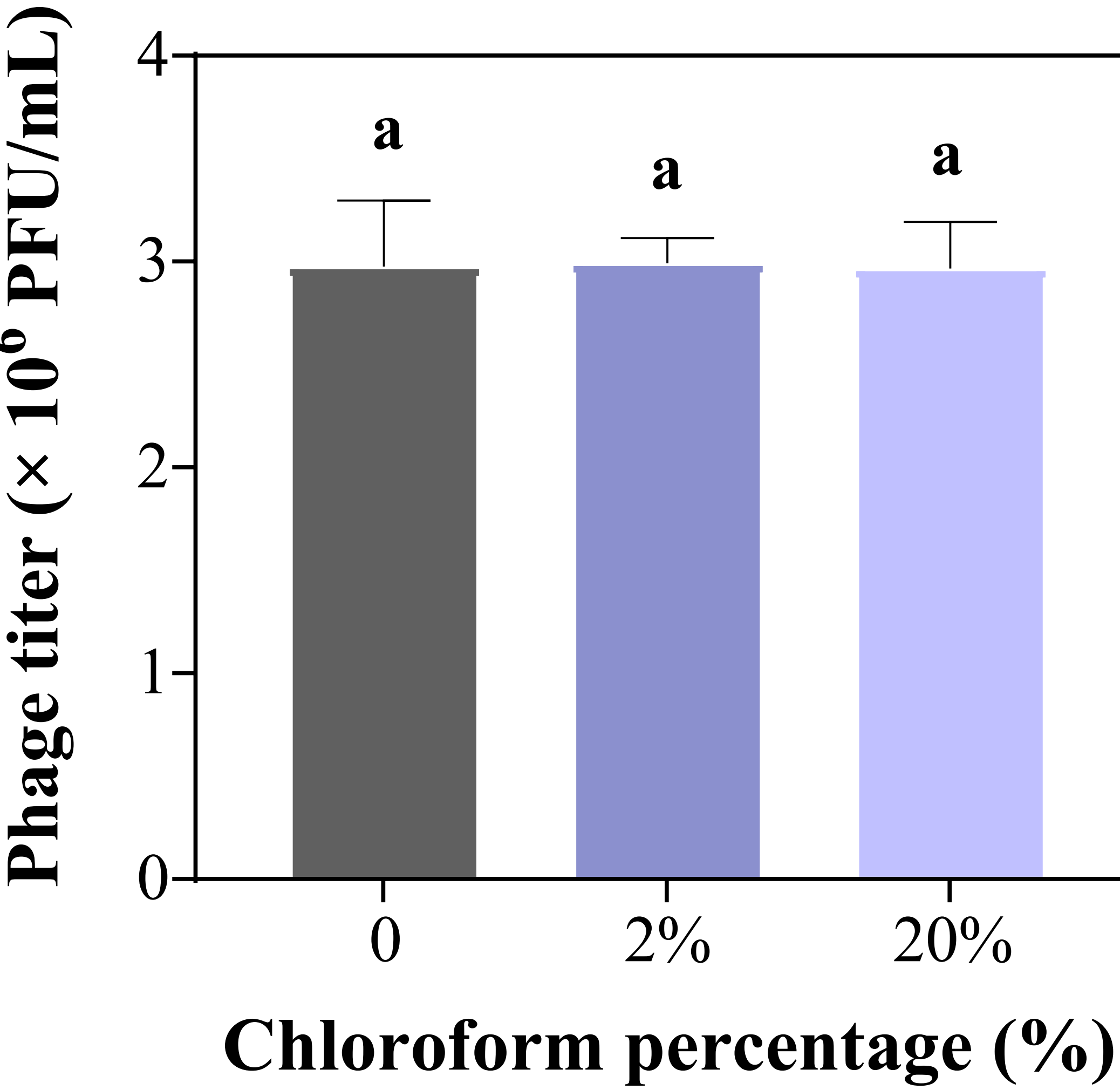

Phage titer (× 10^6 PFU/mL)
4
3
2
1
0
a
a
a
0
2%
20%
Chloroform percentage (%)
