# Supplementary material for: Isolation, characterization, and biocontrol potential of a novel lytic phage vB_VpaS_R27Z against Vibrio parahaemolyticus for mitigating seafood contamination
Source: Curr Res Food Sci. 2026 Oct 22;13:101602. doi: 10.1016/j.crfs.2026.101602 (PMC13737724; doi:10.1016/j.crfs.2026.101602)
Supplement: Supplementary file 2 [file mmc2.pdf]

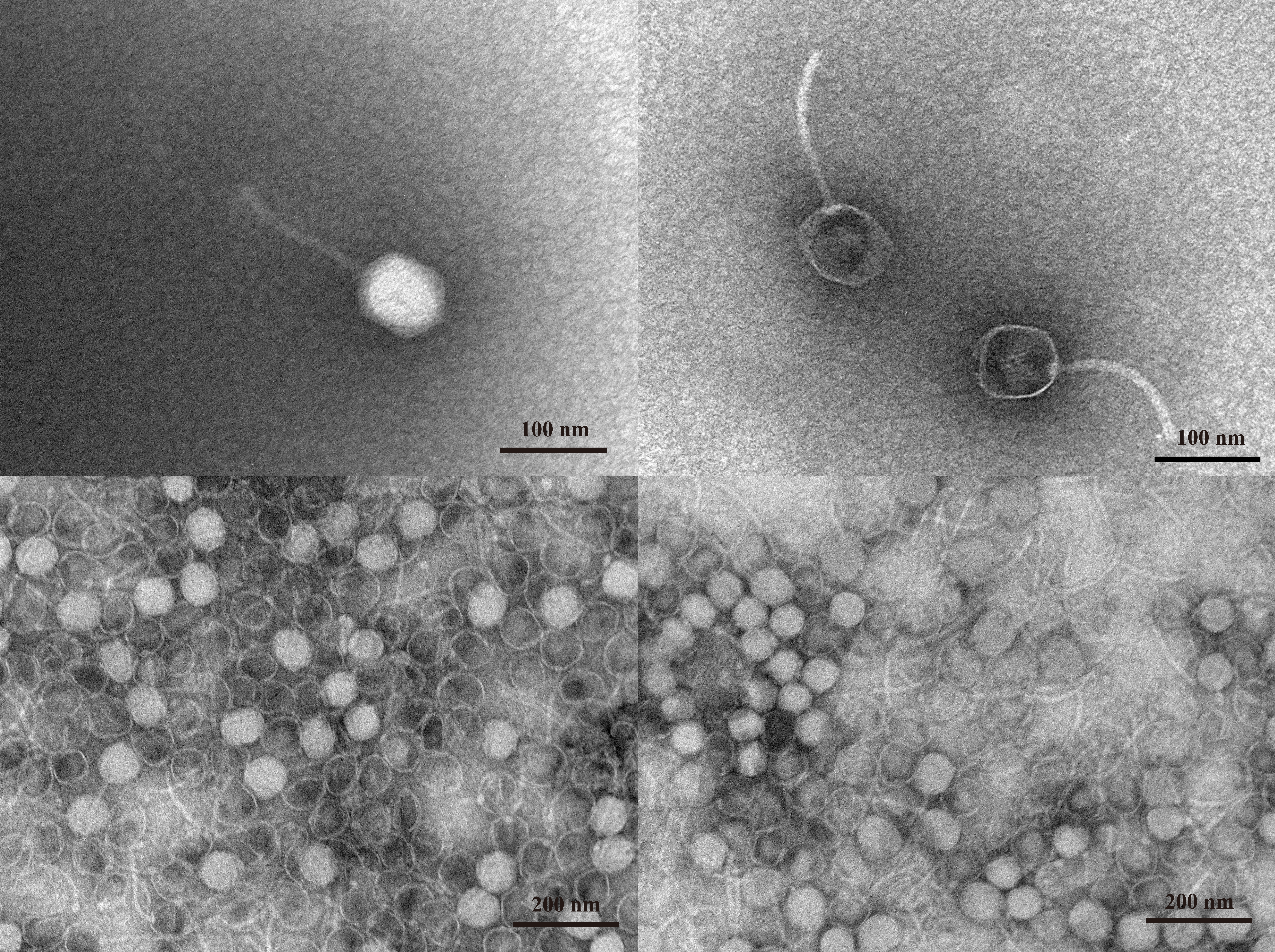

100 nm
100 nm
200 nm
200 nm
